# Tumor Purity in Preclinical Mouse Tumor Models

**DOI:** 10.1158/2767-9764.CRC-21-0126

**Published:** 2022-05-10

**Authors:** Wubin Qian, Xiaobo Chen, Yanghui Sheng, Likun Zhang, Jingjing Wang, Zhenzhen Song, Qi-Xiang Li, Sheng Guo

**Affiliations:** 1 Crown Bioscience Inc., Suzhou, P.R. China.; 2 Crown Bioscience Inc., Taicang, P.R. China.; 3 Crown Bioscience, Inc., Santa Clara, California.

## Abstract

Tumor biology is determined not only by immortal cancer cells but also by the tumor microenvironment consisting of noncancerous cells and extracellular matrix, together they dictate the pathogenesis and response to treatments. Tumor purity is the proportion of cancer cells in a tumor. It is a fundamental property of cancer and is associated with many clinical features and outcomes. Here we report the first systematic study of tumor purity in patient-derived xenograft (PDX) and syngeneic tumor models using next-generation sequencing data from >9,000 tumors. We found that tumor purity in PDX models is cancer specific and mimics patient tumors, with variation in stromal content and immune infiltration influenced by immune systems of host mice. After the initial engraftment, human stroma in a PDX tumor is quickly replaced by mouse stroma, and tumor purity then stays stable in subsequent transplantations and increases only slightly by passage. Similarly, in syngeneic mouse cancer cell line models, tumor purity also turns out to be an intrinsic property with model and cancer specificities. Computational and pathology analysis confirmed the impact on tumor purity by the diverse stromal and immune profiles. Our study deepens the understanding of mouse tumor models, which will enable their better and novel uses in developing cancer therapeutics, especially ones targeting tumor microenvironment.

**Significance:**

PDX models are an ideal experimental system to study tumor purity because of its distinct separation of human tumor cells and mouse stromal and immune cells. This study provides a comprehensive view of tumor purity in 27 cancers in PDX models. It also investigates tumor purity in 19 syngeneic models based on unambiguously identified somatic mutations. It will facilitate tumor microenvironment research and drug development in mouse tumor models.

## Introduction

In patient tumors, cancer cells are embedded in and interact with the surrounding tumor microenvironment (TME) that consists of stroma, infiltrating immune cells, blood vessels, and signaling molecules. TME strongly affects tumor progression, invasion, metastasis, and treatment, and differs between cancers (1, 2). For example, pancreatic ductal adenocarcinoma is characterized by heavy stromal content, in addition to varied TME compositions in its subtypes (3), causing great challenges for drug penetration and access to the cancer cells. Systematic studies have shown that tumor purity, the proportion of cancer cells among all cells in a tumor, varies by cancer and is a distinct feature of cancers (4, 5). For example, it was found that brain-originated cancer has high tumor purity, while pancreatic cancer has low purity. Tumor purity is associated with certain clinical features and outcomes (4). Cancer genomics research is prone to spurious results, if the genomic profiles (e.g., gene expression and mutation) unique to cancer and noncancerous cells are not properly distinguished and handled in the analysis (4, 6–9). Therefore, precise measurement and thorough understanding of tumor purity is important to cancer treatment.

Preclinical mouse tumor models are widely used in studying tumor biology and supporting cancer drug development (10–13). A comprehensive understanding of tumor purity in mouse tumor models will tremendously help model selection, study design, data interpretation, and clinical translation. In patient-derived xenograft (PDX) models, which are widely considered as one of the most predictive models of patient tumors, human tumor fragments are engrafted into immunodeficient mice, and human stroma components are usually quickly replaced by their mouse counterparts (14–16), with the infiltration of mouse immune cells and blood vessels, etc. Tumor purity in PDX models, therefore, is the proportion of human cancer cells in its admixture with mouse noncancerous cells, and can be directly obtained if the ratio of human to mouse cells is known. It is yet unclear how tumor purity changes during successive transplantations of tumors in mice, and whether PDX tumor purity still maintains the cancer specificality seen in patient tumors. There are several classes of immunodeficient mice with varied degrees of immune deficiency. BALB/c nude mice are T-cell deficient, NOD-SCID mice are deficient in T cells and B cells with functional impairment for macrophages, dendritic cells and natural killer cells. Whether host mouse strain impacts tumor purity is another question of interest.

Syngeneic tumor and homograft mouse tumor models, with fully functional immune systems, are also regularly used in immuno-oncology research and immunotherapy development (11, 17). In these models, mouse cancer cell lines or tumor fragments are implanted into inbred immunocompetent mice of the same genetic background. Though commonly used syngeneic cell line models have been extensively studied to reveal their diverse TME and immune profiles (18–21), tumor purity is largely an untouched topic. Analogous to human tumors, tumor purity in syngeneic models is the ratio of mouse cancer cells among the mouse cell population in a tumor, and it can be estimated on the basis of the variant allelic fraction (VAF) of somatic mutations unique to the cell line.

Tumor purity can be estimated for patient tumors by pathology straining with visual morphology inspection as well as by inference from DNA methylation, mutation, copy-number alteration, and gene expression in genomic data, though low concordance frequently exists between different methods (5, 6, 22–25). For PDX models, besides pathology and genomic methods, there are several experimental approaches for separating human and mouse cells to estimate tumor purity, including FACS, antibody-based separation, and PCR assays (26), all with limited sensitivity and accuracy. We recently developed a deep next-generation sequencing (NGS) assay that achieves high accuracy (error <1%) in estimating the ratio of human cells in a mix of human and mouse cells (27), it is a powerful tool for estimating and understanding tumor purity in PDX models (see Materials and Methods, Tumor purity estimation for PDX models using a deep NGS assay). We also developed a maximum-likelihood method for estimating tumor purity in syngeneic models.

Here, we report the first systematic pan-cancer analysis of tumor purity in PDX models using almost 7,000 PDX tumors over 30 cancers that were profiled by the deep NGS assay. We first checked how quickly mouse stroma replaces human stroma in PDX tumors and how stable tumor purity is in successive passages of PDX tumors. We then investigated whether PDX models maintain cancer-specific tumor purity, and whether mouse strain affects tumor purity in NOD/SCID and BALB/c nude mice. We evaluated several methods that estimate PDX tumor purity from whole-exome sequencing (WES) and RNA-sequencing (RNA-seq) data, using tumor purity from the deep NGS assay as ground truth. We were also able to compare TME in PDX tumors between cancers using transcriptome-derived immune and stromal scores. In the second part, we studied tumor purity in 19 syngeneic models, each with five tumors profiled by RNA-seq. We determined that syngeneic tumor purity is model specific, and is associated with tumor immunogenicity, stromal content, and immune infiltration.

## Materials and Methods

### Mouse Tumor Model Establishment

PDX models were developed and established in immunodeficient mice at Crown Bioscience. The cryopreserved or fresh tumor tissues were cut into small pieces (∼2–3 mm in diameter), then subcutaneously transplanted in the right flank of NOD/SCID or BALB/c nude mice for PDX model development. For the development of the syngeneic tumor models, mouse cell lines were mainly obtained from ATCC or other commercial cell centers, and expanded *in vitro* under the culture condition suggested by manufacturers. Cells were harvested when growing in exponential growth phase and subcutaneously engrafted into immunocompetent mice with same genetic background for tumor development. All the animal study procedures were performed in the specific pathogen-free animal facility at Crown Bioscience under the approved protocols by the Institutional Animal Care and Use Committee.

### NGS for Mouse Tumor Models

#### Nucleic Acid Extraction

Snap frozen mouse tumor tissues were powdered in liquid nitrogen. Nucleic acid extraction was based on the methods and reagents described in the operation manuals of RNeasy mini kit (QIAGEN, catalog no. 74104) and DNeasy Blood & Tissue kit (QIAGEN, catalog no. 69504). The integrity of the cleanup RNA was determined by Agilent RNA 6000 Pico chip (Agilent, catalog no. 5067-1513) and 2100 Bioanalyzer (Agilent) and quantified using NanoDrop 2000/2000c Spectrophotometer (Thermo Fisher Scientific). Only high-quality RNA samples (OD260/280 = 1.8–2.2, OD260/230 ≥ 2.0, RNA integrity number (RIN) ≥ 7, >500 ng) were used to construct sequencing library. The integrity of the total DNA was determined by 2100 Bioanalyzer (Agilent) and quantified using NanoDrop 2000/2000c Spectrophotometer (Thermo Fisher Scientific). High-quality DNA samples (OD260/280 = 1.8–2.0, OD260/230 ≥ 2.0, DNA integrity number (DIN) ≥ 7, >500 ng) were used to construct sequencing library.

#### RNA-seq

Library construction for RNA-seq was performed with matched kits following the guide provided by the manufacturer (MGI, catalog no. 1000006383). Briefly, poly-A mRNA was captured and purified from total RNA using Oligo-dT–attached magnetic beads (Agencourt, catalog no. A63987), followed by fragmentation using fragment buffer. cDNA was synthesized following the process: first-strand cDNA synthesis, second-strand cDNA synthesis, and cleanup. End-repair and cleanup were then performed to the purified double-stranded cDNA, followed by A-tailing, ligation of adapters and cleanup. DNA fragments with adapters were selected and amplified by PCR. After PCR product cleanup, the final library was quantified and qualified by Qubit and Agilent 2100 Bioanalyzer again before sequencing. A library with good concentration and fragment size was then proceeded to sequencing. The final library was sequenced following the official guide provided by the manufacturer (MGI, catalog no. 1000012555). The sequencing read length is paired-end PE150.

#### WES

For whole-exome sequencing, DNA library construction and hybrid selection of genomic DNA (gDNA) was performed using the SureSelect XT HS and XT Low Input Human All Exon V7 kit (Agilent, catalog no. 5191-4029). Sequencing was performed on Illumina NovaSeq 6000 with PE150. gDNA was sheared by ultrasonic disruption, followed by end-repair and adding dA-tail to the DNA end, ligation of molecular-barcoded adaptor and library purification. The adaptor-ligated library was amplified and the PCR products were cleaned up using AMPure XP beads. Library quality and quantity was assessed by Qubit and Agilent 2100 Bioanalyzer, and libraries with good concentration and fragment size were then proceeded to hybridization and capture. The SureSelect-enriched DNA libraries were PCR amplified, followed by cleanup. The sequencing libraries were then analyzed by Agilent 2100, high-quality libraries were pooled for multiplexed sequencing. After library validation, Illumina cBOT cluster generation system with Illumina NovaSeq 6000 S4 reagent kit was used to generate clusters. Paired-end sequencing was performed by NovaSeq 6000 following Illumina-provided protocols for PE150 sequencing.

### NGS Data Analysis for Mouse Tumor Models

The quality of raw data was checked by FastQC (version 0.11.9). Adapter and low-quality sequences were trimmed by Trimmomatic software (version 0.40; ref. 28). For PDX samples, sequencing reads were mapped to human (hg19) and mouse (mm10) reference genomes using STAR (version 2.7.10a; ref. 29) for RNA-seq data and BWA (version 0.7.17; ref. 30) for WES data. Reads preferentially mapped to the mouse or human genomes were taken as mouse or human reads, respectively. Ambiguous reads were discarded (Supplementary Fig. S1). Human gene expression was estimated by kallisto (version v0.46.1; ref. 31) from human reads by using ENSEMBL human reference genes (version GRCh38.102). Mouse gene expression was estimated by kallisto from mouse reads by using ENSEMBL mouse reference genes (version GRCm38.101). In addition, we merged the human and mouse reference genes to create a hybrid reference to which we first used kallisto to estimate transcript expression for both human and mouse genes, then aggregated expression of all transcripts of a human gene and its mouse orthologous gene(s) as the hybrid expression for the gene. Nucleotide variants in human genes were detected using GATK best practice workflow (32), which involved read mapping by STAR software, read sorting and duplication marking by Picard software (version 2.25.1, http://broadinstitute.github.io/picard/), read splitting (only for RNA-seq data), INDEL realigning, haplotype calling, variant filtering by GATK software. The variants were annotated by VEP software (33). For syngeneic models, reads from syngeneic tumor tissue and host mouse tissue were not separable, so gene expression and mutations of syngeneic models were the mixed result of both tumor and stroma tissues. The detection steps were same as analyzing mouse reads in PDX samples.

### Identification of Somatic Mutations in PDX Models

As we did not have genomic data of matched normal tissues for PDX tumors, we predicted the germline and somatic mutations using following criteria: (i) If the allele frequencies in dbSNP, gnomAD or ExAC database were all >0.01, the variant was taken as germline mutations (34); (ii) Variants detected in >20% of tested PDX models were taken as germline mutations; (iii) If a variant was found in more than one of other 45 vertebrate genomes, its was taken as germline mutation. The 46-way gene sequence alignment was downloaded from the UCSC database (4, 35). All variants with mutation frequency smaller than 10% were taken as germline mutations. Other variants were treated as somatic mutations.

### Tumor Purity Estimation for PDX Models Using a Deep NGS Assay

We previously developed a deep NGS assay for estimating tumor purity with high sensitivity and accuracy (27). Briefly, we identified 108 100–300 bp homologous segments between human and mouse genomes. Each segment has identical flanking sequences on both ends in the two species so that a common primer pair were designed to amplify the segment, which is divergent enough (31%–97% sequence identities) between human and mouse to enable unambiguous assignment of mapped sequencing reads to a species. Deep NGS with average coverage over 3,000 was used to sequence a PDX tumor, thus the ratio of human and mouse reads was precisely obtained for each segment, and median of all the 108 ratios was taken as the ratio of human and mouse cells in the PDX tumor.

### ESTIMATE Algorithm Applied to Mouse Tumor Models

There are 141 human genes in both the immune and stromal signatures of the ESIMATE algorithm (6), we were able to map 130 and 141 genes to their mouse orthologs, respectively (Supplementary Table S1), based on the orthologous relationship between the two species in the ENSEMBL database (Release 104) retrieved by the biomaRt tool (version 2.50.3; ref. 36). For PDX tumors, we estimated three versions of ESTIMATE scores, each also additionally with an immune score and a stromal score, by the R package *estimate* (version 1.0.13; ref. 6). Specifically, the original *human* version uses only expression of the human signature genes; the *mouse* version uses only the expression of mouse orthologs of human signature genes; while in the *hybrid* version, we used the hybrid expression of the signature genes, described in a previous section, that essentially adds up the expression of a human signature gene and its mouse orthologous gene(s). For syngeneic tumors, there is only a mouse version of ESTIMATE scores. For a PDX tumor, three tumor purities were also calculated from the three ESTIMATE scores as described below.

ESTIMATE scores for 7,098 patient samples from The Cancer Genome Atlas (TCGA) were downloaded from the ESTIMATE website hosted at MD Anderson Cancer Center (https://bioinformatics.mdanderson.org/estimate/, version 2.0.0, release date 2016-01-22). Their tumor purities were previously inferred by the ESTIMATE algorithm (4), and retrieved through the R package *TCGAbiolinks* (version 2.22.4; refs. 37, 38). A nonlinear *loess* (locally estimated scatterplot smoothing) curve was fitted between ESTIMATE score and tumor purity (Supplementary Fig. S2). The three ESTIMATE scores of a PDX tumor were used as input to the *loess* curve, by interpolation or extrapolation, to get three corresponding tumor purities. We stress that the three tumor purities have distinct biological interpretations. The tumor purity by the human version ESTIMATE measures the proportion of cancer cells in the human cells of the PDX tumor; the tumor purity by the mouse version ESTIMATE measures the proportion of cancer cells in the mouse cells of the PDX tumor; the tumor purity by the hybrid version ESTIMATE measures the proportion of cancer cells in all the human and mouse cells of the PDX tumor. As we will show in Results, the latter two lack accuracy.

### Tumor Purity Estimation for Syngeneic Models

For a syngeneic cell line, we compared its DNA sequences from WES with those of its host mouse strain to identify SNPs, which are considered to be somatic mutations to the mouse. To reduce false positives, we excluded all SNPs with read depth smaller than 20. Somatic mutations can be either homozygous or heterozygous. Syngeneic cells are injected into a host mouse to form a tumor, which now contains cancer cells and host mouse stromal and immune cells. Tumor purity is the ratio of cancer cells in the tumor. Let *θ* be the tumor ratio, then (1 − *θ*) is the ratio of host mouse cells. Without loss of generality, we assume that the genotype at a SNP site is *HH* in the host mouse, and is heterozygote *HT* or homozygote *TT* in the syngeneic cell line, where *H* and *T* can be the four nucleotides. Furthermore, for the heterozygote *HT* in the syngeneic cell line, the nucleotide frequency is *H*_1_ and *T*_1_ for the two nucleotides, respectively. In the sequencing data of the tumor, we denote *n*_*H*_ and *n*_*T*_ as the read depths of the two nucleotides *H* and *T*, and *x* = (*n*_*H*_, *n*_*T*_) as the observed data. For a homozygous *TT* in syngeneic cell line, the likelihood for such an observation *x* in the tumor is



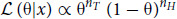



The log-likelihood then is



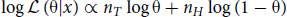



For a heterozygous *HT* in the syngeneic cell line, the likelihood for such an observation *x* in the tumor is



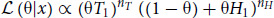



The log-likelihood then is



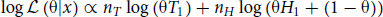



Assume there are *m* observed somatic mutation sites and *X* = (*x*_1_, *x*_2_, …, *x_m_*), the overall log-likelihood is



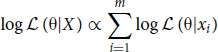



We then solve for *θ* that maximizes the sum above by stepwise increment of *θ* from 0 to 1. In RNA-seq data, SNPs have varied sequencing depths with sometimes magnitude difference. To prevent the distortion of likelihood calculation by high-depth SNPs, we put a depth cap which is five times the median depth.

### Pathology Staining of Tumor Samples

Formalin-fixed and paraffin-embedded tumor samples were deparaffinized with two changes of xylene each for 10 minutes, and subjected to rehydration in two changes of absolute ethanol and three changes of 90%, 80%, and 70% ethanol, each for 2 minutes. After washing with distilled water for 3 minutes, the samples were stained in hematoxylin solution and washed sequentially by distilled water, 0.5% hydrochloric ethanol, distilled water, 0.5% ammonia water and distilled water, then counterstained in eosin and washed by distilled water, dehydrated through 80% and 90% ethanol before cleared in two changes of xylene and mounted with xylene-based mounting medium for scanning. Picrosirius red staining used Picrosirius Red staining kit and phosphomolybdic acid hydrate (HedeBio, catalog nos. 26357-02 and 20029916). CD45 staining used rabbit mAb (Cell Signaling Technology, catalog no. 70257) as primary antibody, and Bond Polymer Refine Detection (Leica, catalog no. DS9800) as secondary antibody detection kit.

### Data Availability Statement

Raw NGS data in FASTQ format were deposited to the Sequence Read Archive with accession number SRP356758 (https://trace.ncbi.nlm.nih.gov/Traces/sra/?study=SRP356758). Data description is provided in Supplementary Table S2. Detailed PDX and syngeneic model information is freely available, with registration, in two databases: https://hubase.crownbio.com and https://mubase.crownbio.com. Data files and R scripts for analysis and graph generation are available at https://github.com/guosheng437/TumorPurity.

## Results

### Tumor Purity in PDX Models

#### Human Stroma is Rapidly Replaced by Mouse Stroma After Initial Implantation

**FIGURE 1:**
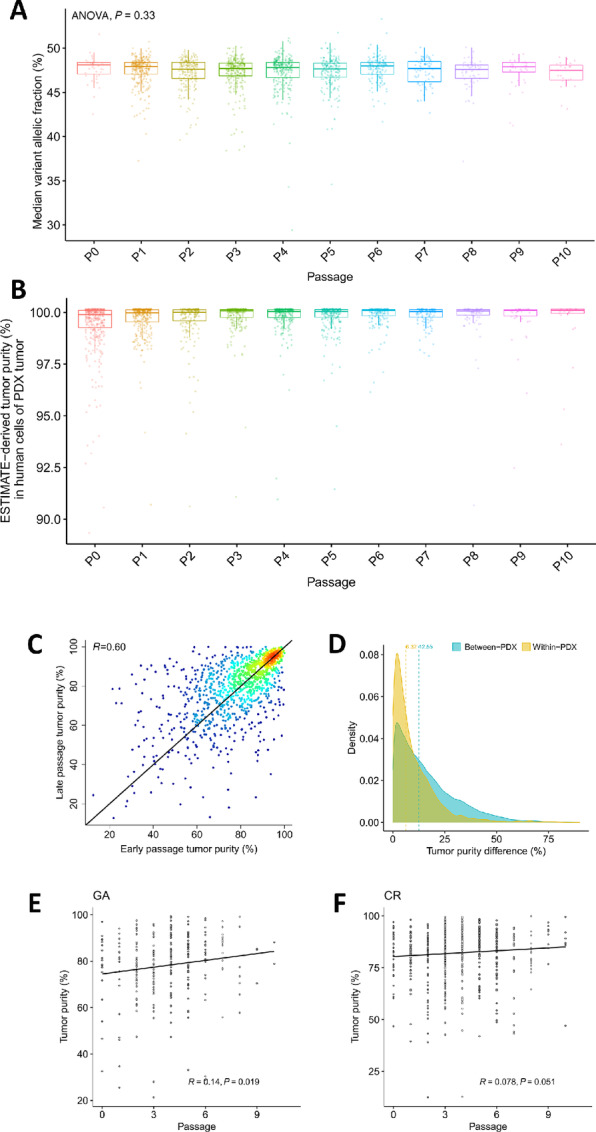
Tumor purity by passage in PDX models. **A,** Median VAF in PDX models by passage. A total of 1,608 PDX models spread into 11 passages. No significant difference was found across passages based on one-way ANOVA. **B,** Tumor purity in the human cells of PDX models by passage. **C,** A strong correlation for tumor purity exists between early and late passages of same model for 1,141 PDX models. **D,** Tumor purity difference between two identical-passage samples from same PDX model and two different PDX models shows that the between-PDX tumor purity difference is much higher than the within-PDX tumor purity difference. Dotted lines are the median tumor purities in the two groups. **E** and **F,** Tumor purity slightly increases by passage in gastric (GA) and colorectal (CR) cancers. The trend may not be statistically significant due to large variation of tumor purity within a passage.

A PDX model is initially established via successful engraftment of a patient tumor fragment in an immunodeficient mouse to form a xenograft tumor of passage P0, which, when reaching sufficient size, say 1,000 mm^3^, is harvested and sliced into smaller fragments for subsequent serial transplantations to form passages 1, 2, 3 and beyond. PDX tumors mimic human tumors in histopathology and genomics (12, 39, 40). We performed WES for a collection of PDX models within passage 10, then computationally identified high-confidence missense somatic mutations. There are 1,608 PDX models each with at least 30 such mutations. We estimated the VAF for each mutation and obtained the median VAF for every model. All 11 passages have comparable median VAFs (one-way ANOVA *P* value = 0.33; Fig. 1A), and are all centered around 47%, the median frequency of clonal monoallelic single-nucleotide variations (which include missense mutations) observed in human pure normal samples and pure tumor samples (25, 41), the slight deviation from 50% is caused by sequencing read mapping bias and other technical biases.

Such concordance indicates that human cells in PDX tumors are completely or near completely cancer cells, that is, human stroma has been replaced by mouse stroma. Results from cancer cell lines and patient tumors support this conclusion. In cancer cell lines, the median VAF is close to 47% in most cancer types, though some cancers, notably breast and esophageal cancers, have lower values possibly due to low-frequency subclonal mutations (Supplementary Fig. S3A). In contrast, median VAFs in patient tumors are much lower mainly because cancer cells are surrounded by non-cancerous immune and stromal cells that do not carry somatic mutations (Supplementary Fig. S3B).

We then estimated the fraction of cancer cells (i.e., tumor purity) in the *human* cells of a PDX tumor using the ESIMATE algorithm (original human version, details described in Materials and Methods) for 2,115 PDX tumors within passage 10 (Fig. 1B). As expected, tumor purity is lowest in P0 because there is still some residual human stroma. After about one to two passages (P1 and P2), tumor purity stabilizes to virtually 100%. Across all passages, 99%, 97.3%, and 96.5% of PDX tumors have tumor purity >95%, 97.5%, and 98%, respectively, in the human cells of PDX models (Supplementary Fig. S4).

Therefore, we conclude that in PDX models, human stroma is quickly and virtually completely replaced by mouse stroma after the initial engraftment. So, we can simply measure the ratio of human cells in the human and mouse cell mix, and use this ratio as the ground truth PDX tumor purity, which is done by a deep NGS assay (27).

#### PDX Tumor Purity is Tumor Specific and Slightly Increases During Passaging

Using our deep NGS assay, we obtained ground truth tumor purities for 6,039 PDX tumor samples harvested from NOD/SCID mice and 770 samples from BALB/c nude mice. In the NOD/SCID collection, there are 1,141 PDX models each with at least two tumor samples. For every model, we randomly selected an early passage sample and a late passage sample to compare their tumor purities, and observed a high concordance (Pearson correlation *R* = 0.60; Fig. 1C).

We next selected all pairs of samples with identical passages from same PDX models and calculated their tumor purity difference. Similar calculation was done for all pairs of samples also with identical passages but from different PDX models. Tumor purity difference from the latter is considerably higher than the former (Fig. 1D; median: 12.55 vs. 6.32, Mann–Whitney test *P* value < 2.2e–16). This distinction is preserved in individual cancers as well (Supplementary Fig. S5). Thus, tumor purity is an intrinsic property of PDX tumors.

Overall, late passage tumors have slightly higher tumor purity than early passage tumors in the 1,141 PDX models (mean ± SD = 1.13% ± 14.3%, paired *t* test *P* value = 0.0039). There are 10 cancers with reasonably large sample sizes for many passages in the NOD/SCID collection, enabling us to examine the change of tumor purity during passaging, which confirmed a slight increasing trend by passage in all 10 cancers (Fig. 1E and F; Supplementary Fig. S6), at least part of the ascending is from the replacement of human stroma by mouse stroma by passage, especially in the early few passages (Fig. 1B). However, tumor purity at any passage within a cancer exhibits great variation that reflects tumor heterogeneity, so that only a weak and sometimes non-significant correlation exists between tumor purity and passage in individual cancers.

#### PDX Tumor Purity is Mouse Strain Dependent

**FIGURE 2:**
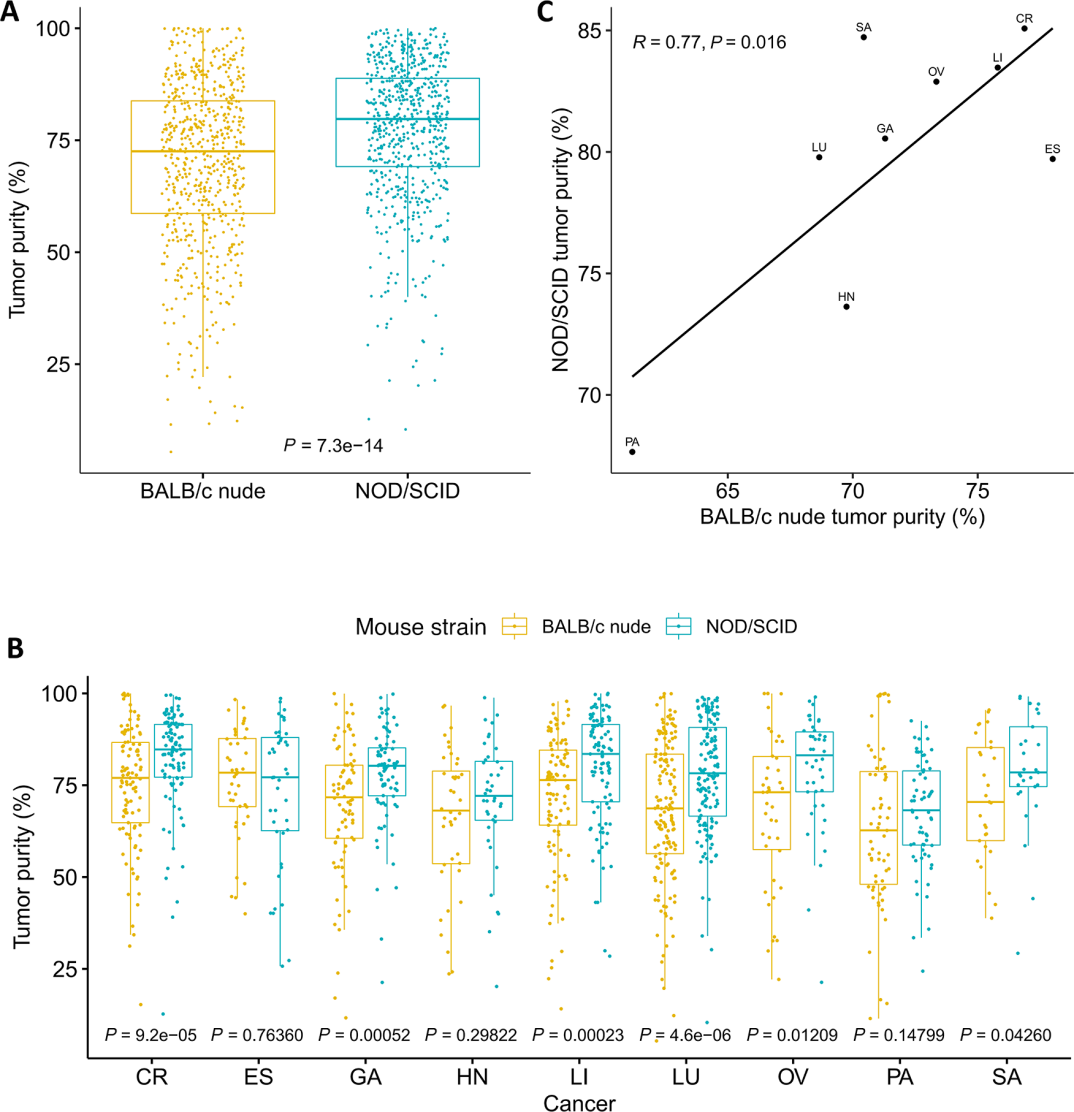
Tumor purity in PDX models depends on mouse strain. **A,** Tumor purity is higher in NOD/SCID than BALB/c nude mice. Tumor purity was calculated using a set of 630 tumor samples in nine cancers in both strains. *P* value is from Mann–Whitney *U* test. **B,** Tumor purity comparison by mouse strain for nine cancers. *P* values are from Mann–Whitney *U* test. **C,** Tumor purity is positively correlated between NOD/SCID and BALB/c nude mice for nine cancers. CR, colorectal; ES, esophageal; GA, gastric; HN, head and neck; LI, liver; LU, lung; OV, ovarian; PA, pancreatic; SA, sarcoma.

We used only NOD/SCID samples in the above analysis because the BALB/c nude samples are far fewer. But more importantly, these two mouse strains have different immune deficiency thus different tumor-infiltrated leukocyte compositions in TME, which may impact tumorigenicity and tumor growth, and therefore should be treated separately. To examine whether mouse strain also affects tumor purity, we compiled a 1,260-sample set with 630 samples for either strain. The samples were from nine cancers and every cancer had the same number of samples in a strain. Interestingly, tumor purity in NOD/SCID mice is significantly higher than BALB/c nude mice in the pooled samples (median: 80.0 vs. 72.6, Mann–Whitney *U* test *P* value = 7.3e–14; Fig. 2A), as well as in individual cancers (Fig. 2B). This observation could indicate that human tumor can grow rather easier in NOD/SCID mice which have less antitumor immunity, thus requiring less stromal component to support its growth. It has been well reported that NOD/SCID mice offer advantages over BALB/c nude mice in growing human tumors (42), and in practice, NOD/SCID mice are preferred to grow thawed xenograft tumors for generating live models. In addition, a high correlation of tumor purity by cancer exists between the two mouse strains (Pearson correlation *R* = 0.77; Fig. 2C). Taken together, it is evident that tumor purity is mouse strain dependent, and its cancer specificity is well preserved in both strains.

#### PDX Tumor Purity is Cancer Specific and Mimics Patient Tumors

**FIGURE 3:**
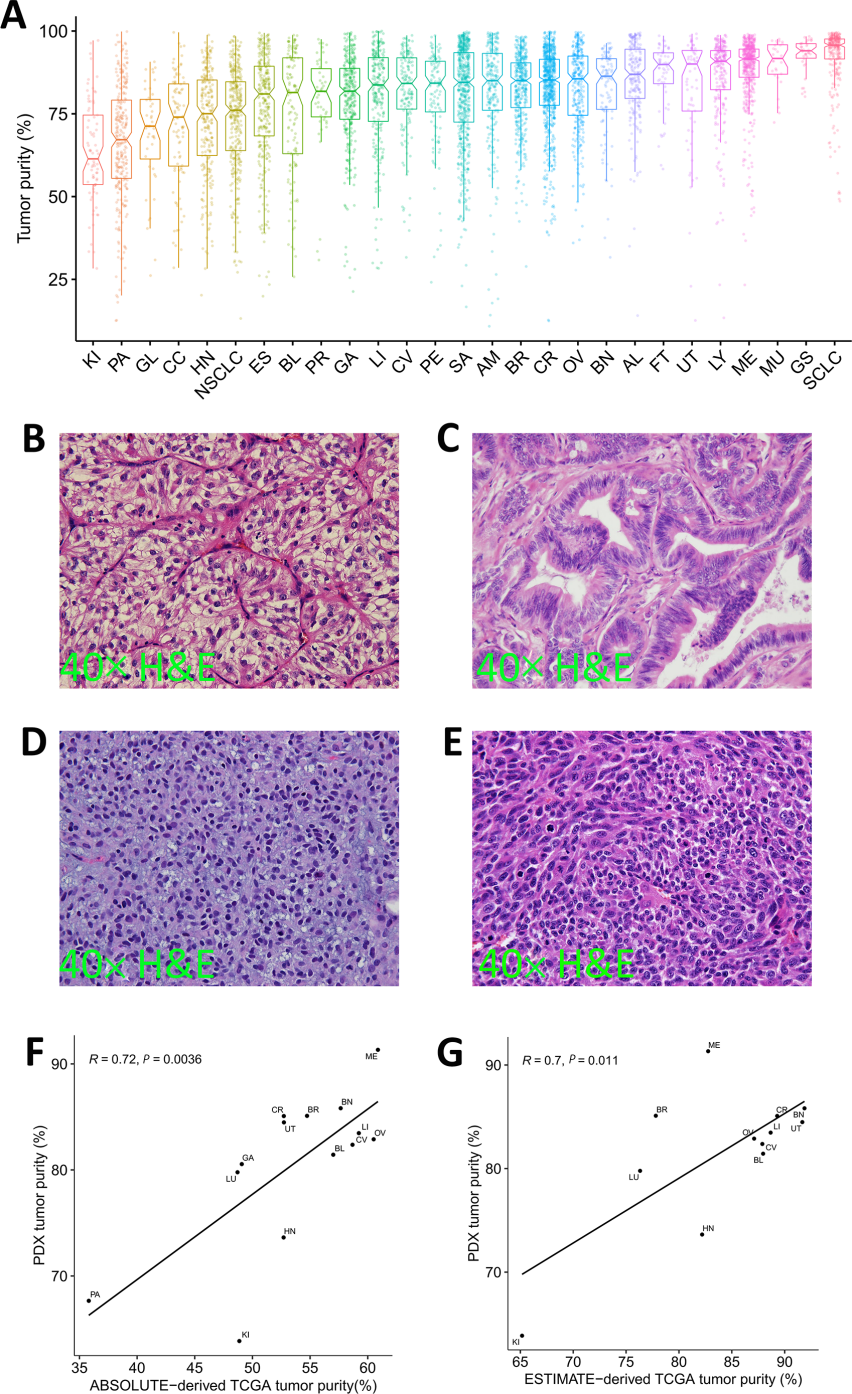
Tumor purity in PDX models is cancer specific and correlates with patient cancers. **A,** Tumor purity in 27 cancers estimated from a deep NGS assay. Hematoxylin and eosin (H&E) staining of PDX tumors in two cancers with low tumor purity: pancreatic ductal adenocarcinoma (**B**) and clear-cell renal cell carcinoma (**C**), and two cancers with high tumor purity: gastrointestinal stromal tumor (**D**) and melanoma (**E**). **F** and **G,** Tumor purity is positively correlated between PDX tumors and TCGA patient tumors. TCGA tumor purity was inferred by the ABSOLUTE method based on somatic DNA alternations (**F**, data from ref. 25), or by the ESTIMATE method based on gene expression (**G**, data from ref. 38). *Cancer abbreviations*: AL, acute lymphoblastic leukemia; AM, acute myeloid leukemia; BL, bladder; BN, brain; BR, breast; CC, cholangiocarcinoma; CR, colorectal; CV, cervical; ES, esophageal; FT, fallopian tube carcinoma; GA, gastric; GL, gallbladder; GS, gastrointestinal stromal tumor; HN, head and neck; KI, kidney; NSCLC, non–small cell lung cancer; LI, liver; LY, lymphoma; ME, melanoma; MU, mixed Mullerian cancer; OV, ovarian; PA, pancreatic; PE, peritoneal; PR, prostate; SA, sarcoma; SCLC, small- cell lung cancer; UT, uterine.

We generated tumor purity distribution in 27 cancers, each with at least 20 samples in the NOD/SCID collection in the early 11 passages (Fig. 3A). The average of median tumor purities across all cancers is 84.5%, and a wide range of tumor purities are seen across cancers. Several cancers have much lower purity than others, including kidney, pancreatic, gallbladder cancers, cholangiocarcinoma, head and neck cancer, and non–small cell lung cancer (NSCLC), all with median purity well below 80%. Kidney cancer has the lowest median purity of 61.4%. Clear-cell renal cell carcinoma, which accounts for about 75% of all kidney cancers (43), is the dominant subtype in our collection. Notably, patient tumors of clear-cell renal cell carcinoma have been found to be of low purity as well (4). Pancreatic cancer also has fairly low tumor purity with a median of 67.4%, consistent with the known fact that it is highly fibrotic. Cholangiocarcinoma occurs in bile duct that connects liver to gallbladder but has lower purity than liver cancer (median: 74.0% vs. 83.7%, Mann–Whitney test *P* value < 5.0e–7), so is gallbladder cancer compared with liver cancer (median: 71.3% vs. 83.7%, Mann–Whitney test *P* value < 2.2e–5). On the other hand, many cancers have very high tumor purity, in particular, small cell lung cancer (SCLC), gastrointestinal stromal tumor, melanoma, and lymphoma all have >90% median purities. The diverse tumor purities are also revealed by pathology staining (Fig. 3B–E).

We next compared tumor purities between PDX tumors and TCGA patient tumors and observed a high positive correlation by cancer for both ESTIMATE (6) and ABSOLUTE (22, 25) inferred patient tumor purities (Fig. 3F and G). Here we used the ground truth tumor purity from the deep NGS assay for PDX tumors, and two computationally inferred tumor purities for patient tumors. The ABSOLUTE method is based on VAF of somatic mutations. In general, PDX tumor purity is comparable with the ESTIMATE-inferred patient tumor purity, but is much higher than the ABSOLUTE-inferred one. The discrepancy comes from the difference of the two computational methods which has been reported before (25): tumor purity from ESTIAMTE is about 50% higher than that from ABSOLUTE. In conclusion, tumor purity in PDX models is cancer specific and faithfully captures cancer specificity in patient tumors.

#### PDX Tumor Purity Estimation from Different Genomics Platforms

**FIGURE 4:**
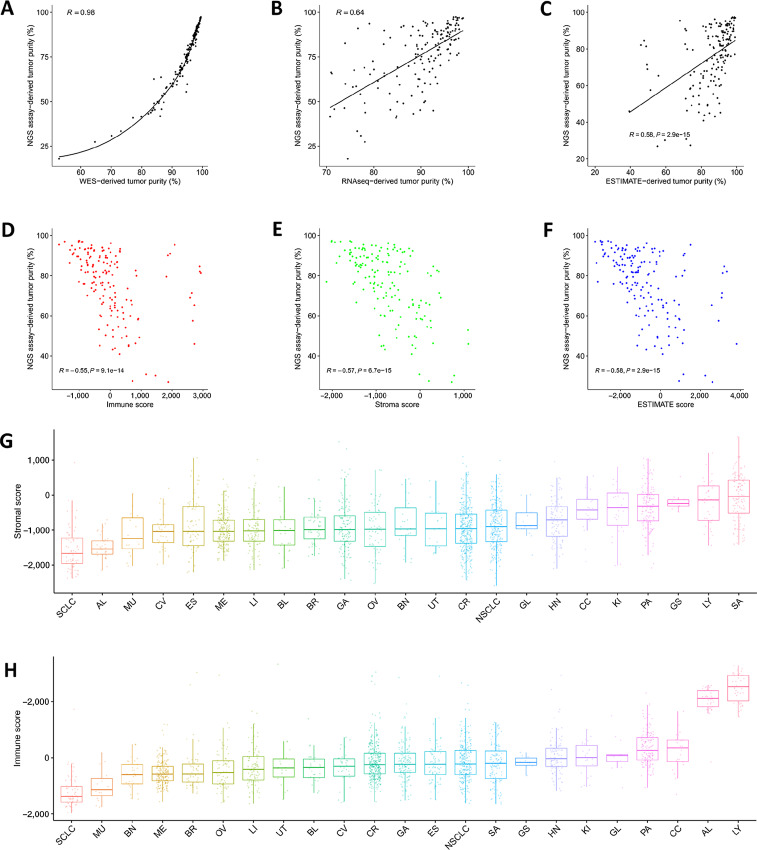
Estimating PDX tumor purity from NGS data. In 157 NOD/SCID PDX tumors, the correlation between the ground truth tumor purities estimated by a deep NGS assay and tumor purities estimated by counting human and mouse reads in WES (**A**) and RNA-seq data (**B**), the former shows a strong nonlinear relationship. **C–F,** A hybrid ESTIMATE algorithm was used to calculate an immune score, a stromal score, an ESTIMATE score for a PDX tumor, the ESTIMATE-derived tumor purity was then calculated. The correlations between the ground truth tumor purity and the four quantities from the hybrid ESTIMATE algorithm were shown. **G** and **H,** Stromal and immune scores of PDX tumors in 23 cancers. Higher score means stronger presence of stromal and immune cells.

Many well-characterized PDX models have been genomically profiled. Here, we set to estimate tumor purity from NGS data using 157 NOD/SCID tumor samples simultaneously profiled by WES and RNA-seq. Their true tumor purities are known from our deep NGS assay. Specifically, we calculated tumor purity from WES and RNA-seq data by first counting 150 bp sequencing reads mapped uniquely to mouse or human genomes, then obtaining the proportion of human reads in the total reads. Like previous reports (27, 44), we observed a nice nonlinear relationship between the true tumor purity and the WES-derived purity (Spearman correlation = 0.98; Fig. 4A), with the latter grossly overestimating tumor purity, which may be used for bias correction. RNA-seq–derived tumor purity, on the other hand, has a much poorer correlation with true tumor purity (Spearman correlation = 0.64; Fig. 4B), which reflects the heterogeneous temporospatial nature of TME gene expression.

Instead of using all genes to estimate tumor purity the ESTIMATE algorithm uses two gene expression signatures. The stromal signature consists of 141 genes with differential expression between tumor and stroma; the immune signature also has 141 genes associated with a variety of immune cells. They are used to detect infiltration of stromal and immune cells in tumor tissues by giving a stromal score and an immune score, lower scores indicate lower infiltration; their sum, the ESTIMATE score, is then used to estimate tumor purity (6). The ESTIMATE algorithm was initially designed for human samples, for convenience, we label it the human version. We previously applied the human version ESTIMATE algorithm to show that PDX tumors essentially have human cancer cells and mouse stroma (Fig. 1B). We next developed a mouse version ESTIMATE algorithm to estimate tumor purity in mouse stroma, which did give very low purity values for the 2,115 PDX tumors analyzed before, albeit significantly higher than the theoretical zero value (Supplementary Fig. S7), a reflection of the algorithm's cross-species limitation.

We also designed a hybrid version ESTIMATE algorithm that takes into account both human and mouse cells in a PDX tumor by aggregating human and mouse orthologous genes in the stromal and immune signatures (details described in Materials and Methods). We observed a good correlation between the true tumor purity from the NGS assay and ESTIMATE score, immune score, stromal score, and tumor purity from the hybrid ESTIMATE algorithm (Fig. 4C–F). The hybrid ESTIMATE algorithm tends to overestimate tumor purity in PDX models.

#### PDX Tumors Differ in Stromal Content and Infiltrated Immune Cells

We probed TME of PDX tumors using stromal and immune signatures from the hybrid version ESTIMATE algorithm, and observed large variance across cancers (Fig. 4G and H). It is not surprising to see three non-epithelial cancers—gastrointestinal stromal tumor, sarcoma and lymphoma—have the highest stromal content, followed by pancreatic cancer with lots of tumor-associated fibroblasts and kidney cancer. SCLC is very distinct in that it has the lowest infiltrated stromal and immune cells and highest tumor purity, in contrast to NSCLC. Acute lymphoblastic leukemia, a hematological malignancy, has expectedly low stromal content but strong immune characteristics. Lymphoma develops from lymphoid lineage and consequently has the highest immune infiltration. Other cancers have significantly lower immune infiltration, particularly SCLC and mixed Mullerian cancer.

### Tumor Purity in Syngeneic Models

Syngeneic mouse tumors grow in immunocompetent mice of same inbred genetic background. Unlike PDX tumors, it is difficult to separate cancer and noncancerous cells in syngeneic tumors either experimentally or *in silico*, because all cells are of mouse origin, a similar situation as patient tumors. On the other hand, it is straightforward to identify somatic mutations since they are the ones solely carried by the syngeneic cell lines. We developed a maximum-likelihood algorithm to accurately estimate tumor purity in syngeneic tumors from RNA-seq data, and applied it to 19 syngeneic models, each with five tumors harvested at around 500 mm^3^ and followed by dissociation and homogenization before sequencing. Syngeneic models harbor remarkably different numbers of somatic mutations (Table 1). 4T1, J558, and RM1 all have fewer than 100 somatic mutations and are known to be poorly immunogenic (45), while some other syngeneic models, such as H22 and MC38, carry a lot more somatic mutations and are very immunogenic. There appears to be no correlation between tumor purity and the number of somatic mutations (Spearman correlation = 0.16, *P* = 0.50).

**TABLE 1:** Tumor purity and number of somatic mutations in 19 syngeneic models.

Syngeneic model	Tumor purity (%)^a^	Number of somatic mutations	Cancer
Pan02	78	204	Pancreatic cancer
MBT2	78.7	1,194	Bladder cancer
EMT6	80.6	214	Breast cancer
LL2	81.7	1,741	Lung cancer
WEHI164	83.4	848	Fibrosarcoma
4T1	83.6	66	Breast cancer
Renca	84.8	654	Kidney cancer
RM1	87.2	60	Prostate cancer
MC38	88	1,662	Colorectal cancer
CT26	88.2	1,027	Colorectal cancer
EL4	89.5	879	Lymphoma
EG7OVA	90.6	563	Lymphoma
A20	92.1	320	Lymphoma
B16F10	94.3	458	Melanoma
Colon26	94.3	783	Colorectal cancer
H22	95.3	3,447	Liver cancer
J558	96	75	Plasmacytoma
B16BL6	96.3	559	Melanoma
KLN205	96.3	532	Lung cancer

^a^Median value of five samples dissected at around 500 mm^3^.

**FIGURE 5:**
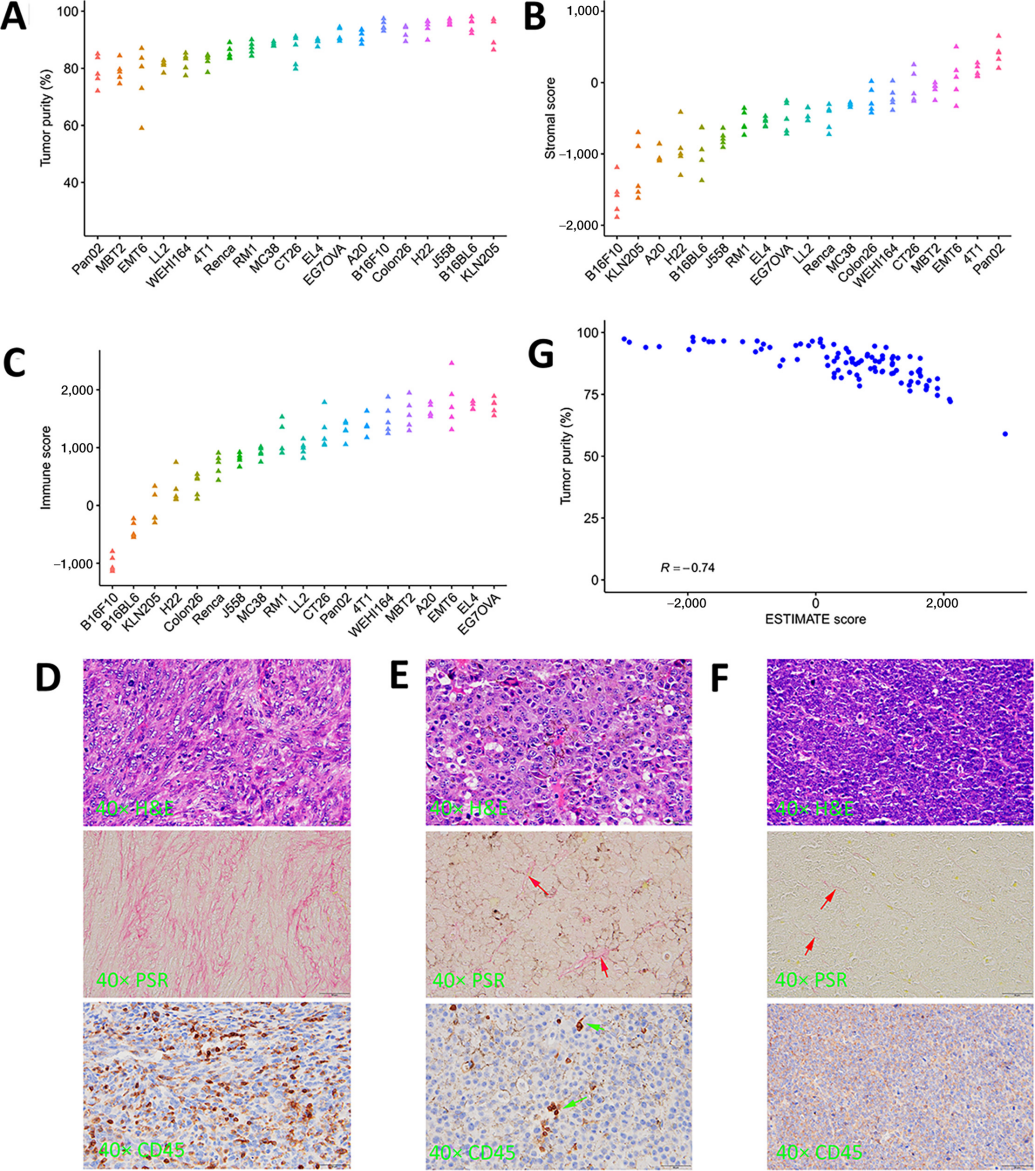
Tumor purity in syngeneic models. **A,** Tumor purity in 19 syngeneic models. Each model has five tumor samples harvested at about 500 mm^3^ from immunocompetent host mice. **B** and **C,** Stromal and immune scores of the 19 models. Higher score means more stroma or stronger immune infiltration in the tumor. **D,** Correlation between tumor purity and the mouse version ESTIMATE score, which was calculated by replacing human signature genes by orthologous mouse genes. Pathology of three syngeneic models: Pan02 (**E**), B16F10 (**F**), and A20 (**G**), with (top) H&E staining, (middle) picrosirius red staining, and (bottom) CD45 IHC staining. Red arrows point to collagen fibers and green arrows to CD45^+^ cells.

In general, tumor purity varies by 10%–20% within a model, suggesting that it is a relatively stable feature of syngeneic models (Fig. 5A). We emphasize here that sample collection and processing is critical to get unbiased assessment of tumors in bulk sequencing, and the whole-tumor homogenization approach should be used. In studies where tumors were sliced and some random fragments were used for sequencing, we frequently observed very high or low tumor purity, for example, one B16BL10 tumor had an exceptionally low 52% tumor purity.

Syngeneic models differ in tumor purity, ranging from approximately 80% to 96%. We examined TME in these models using a mouse version ESTIMATE algorithm that substitutes all human signatures genes by mouse orthologs to calculate a stromal score and an immune score for a tumor (details described in Materials and Methods). Pancreatic cancer model Pan02 registered the lowest tumor purity with the highest stromal score and a high immune score (Fig. 5B and C), which was confirmed by pathology staining (Fig. 5D). It seems that high tumor purity comes from reduced stromal content as well as suppressed immune infiltration. B16F10, a melanoma model, has the lowest immune and stromal scores (Fig. 5B and C). Indeed, only trace amount of collagen fibers and sporadic CD45^+^ cells were observed in its pathology staining (Fig. 5E). B16BL6, a close relative of B16F10, exhibits a similar pattern, so do KLN205 and H22. Three lymphoma models (A20, EL4, EG7OVA) expectedly have very high immune content but varied stromal content. In particular, A20 has a much lower stromal score (Fig. 5B) verified by picrosirius red staining (Fig. 5F). Both breast cancer models, 4T1 and EMT6, have very high immune and stromal content. Therefore, TME including tumor purity in syngeneic models is both model and cancer specific.

Finally, we evaluated the relationship between tumor purity and mouse version ESTIMATE score for all 95 syngeneic tumors. A significant yet imperfect negative correlation exists between the two variables (Pearson correlation *R* = −0.74; Fig. 5G). Species difference undoubtfully contributes to such deterioration, since mouse immune system is different from human. Yet it also demonstrates, in conjunction with the PDX cases, that gene expression methods for estimating tumor purity have relatively large errors for mouse models. Instead, more rigorous methods, like the maximal-likelihood method we developed here, should be preferred as a routine procedure in analyzing syngeneic tumors both before and after drug treatment for tumor purity and TME studies.

## Discussion

Tumor purity has been extensively studied in patient tumors but never been systematically investigated in preclinical mouse tumor models. This study fills the gap. Study of tumor purity in patient tumors has been hampered by the practical difficulty of separating cancer cells from noncancerous cells by experimental approaches, particularly so for excluding normal epithelial cells in carcinomas. Considerable discordance exists between different inference methods, be it pathology analysis or NGS-based inference (5, 25). In contrast, preclinical mouse tumor models are ideal systems to study tumor purity because it can be accurately estimated from NGS data by computational and statistical methods due to species distinction in PDXs and known somatic mutations in syngeneic models.

We demonstrated that in PDX models, tumor purity is cancer specific, agrees well with patient cancers, and shows large variations across cancers. Given the rapid and virtually complete replacement of noncancerous human cells by mouse counterparts in initial implantation of patient tumors in host mice, it is most plausible that tumor purity is determined by cancer cells *per se* via their interactions with TME. This postulation is supported by the observation that early and late passage tumors of same PDX model have highly similar and correlated tumor purities, and also by results from syngeneic models in which dissociated pure mouse cancer cells are injected into host mice to form tumors that also exhibit model and cancer specific tumor purity. The importance of TME in influencing tumor purity is further revealed by the comparative study in two immunodeficient mouse strains: tumors in the less immune impaired BALB/c nude mice have significantly more noncancerous cells thus lower tumor purity than in NOD/SCID mice.

Tumor heterogeneity, in terms of the spatial composition of tumor, is another source of tumor purity variation. PDX and syngeneic tumors maintain histological structures like patient tumors, where different regions of a tumor can have different proportions of cancer cells (4). We tried to minimize the influence of such heterogeneity in the analysis of 19 syngeneic models by first dissociating whole tumors harvested at around 500 mm^3^, then homogenizing all cells before taking a portion for RNA-seq. We did see relatively small variance of tumor purity in nearly all 19 syngeneic models (Fig. 5A), even though the five samples of a model came from 5 different mice. The variance mostly resulted from intrinsic difference of tumor TME and some randomness introduced in experiments.

Studies on patient tumors, as well as ours in mouse tumor models, clearly demonstrated that tumor purity is an intrinsic property of cancer. Furthermore, quantities of stromal and infiltrating immune cells are also cancer and tumor specific. It is therefore critical to take into account the noncancerous mouse cells to gain a comprehensive understanding of PDX tumors, a practice commonly neglected in genomic analysis on PDX. In this work, we showed that merging human and mouse orthologous genes gave better tumor purity estimation by the gene expression-based ESTIMATE method (Fig. 1B and 4C). Another example is for the classification of colorectal cancers in PDX models. Patient colorectal cancers have been classified into four consensus molecular subtypes (CMS; ref. 46). CMS4 is a mesenchymal subtype with strong stromal invasion and angiogenesis. CMS4 is only recapitulated in PDX models when mouse stromal gene expression is incorporated (47). Similarly, syngeneic models all have characteristic TME, manifested as heterogeneous gene expression for immune cells, phenotypically inflammatory or noninflammatory, so that the selection of proper models for assessing immunotherapeutic drugs needs fairly comprehensive characterization of TME, often times by NGS and microarray profiling, flow cytometry experiment and cytokine quantification (20).

Many computational methods have been developed to infer tumor purity from NGS data. In this report, we performed a multifaceted assessment of the gene expression–based ESTIMATE approach, and showed its capability of quantifying immune and stromal contents in tumors, and its limitation in estimating tumor purity. We proposed a maximum-likelihood method for estimating tumor purity for syngeneic models using NGS data. We would like to emphasize that this approach can be applied to human tumors if there are matched normal tissues with NGS data as well. Specifically, we first identify SNPs that are homozygous in the normal tissue but heterozygous in the tumor, then use the maximum-likelihood method to estimate the proportion of normal cells in the tumor, and subsequently get tumor purity.

A limitation of this study is the lack of in-depth characterization of TME in both PDX and syngeneic models, which would need extensive experimental approaches such as pathology staining, FACS and single-cell sequencing, somewhat impractical for our 7,000 tumors tested by the deep NGS assay, which can only quantify tumor purity. We tried to estimate immune and stromal contents in tumors from RNA-seq data, much the same way for analyzing TCGA patient tumors (4), and performed comparative studies between mouse model tumors and patient tumors. We understand that TME is not only cancer specific but tumor unique, and is a more complex subject than tumor purity.

The prevailing existence of mouse stromal and immune cells in PDX tumors, particularly so for cancers with low tumor purity, warrants the need to separate human and mouse cells for studies that can be affected or impeded by their mixing, such as proteomic profiling of PDX tumors, in which correct peptide assignment to human or mouse is impractical from the mass spectrometry readouts of whole PDX tumors, so accurate quantification of tumor and stromal specific proteins is possible only by separating them prior to proteomic profiling. Unfortunately, this was often overlooked in many PDX proteomics studies.

In conclusion, our work gives a bird's-eye view of the complex tumor purity landscape in preclinical mouse tumor models. It provides another layer of evidence on the resemblance of xenograft and homograft tumors to patient tumors, but also unveil the uniqueness and difference of mouse tumor models, so that extra caution must be exercised in understanding, selecting, and using mouse models for the development of more effective therapeutics to treat cancer.

## Supplementary Material

Supplementary Tables 1-2 Table S1-S2

Supplementary Figure 1 Computational workflow for mapping RNAseq and WES sequencing reads to human and mouse genomes.

Supplementary Figure 2 Tumor purity inferred from ESTIMATE score from RNAseq data for human samples.

Supplementary Figure 3 Median variant allelic fraction (VAF) of missense mutations in cancer cell lines and patient tumors.

Supplementary Figure 4 The distribution of tumor purity in human cells of 2115 PDX models within passage 10.

Supplementary Figure 5 Within- and between-PDX tumor purity difference for 18 cancers.

Supplementary Figure 6 PDX tumor purity change by passage for 8 cancers.

Supplementary Figure 7 The distribution of tumor purity in mouse cells for 2115 PDX models within passage 10.
