# Supplementary material for: Tumor Purity in Preclinical Mouse Tumor Models
Source: Cancer Res Commun. 2022 May 10;2(5):353–65. doi: 10.1158/2767-9764.CRC-21-0126 (PMC9981214; doi:10.1158/2767-9764.CRC-21-0126)
Supplement: Supplementary Figure 1 — Computational workflow for mapping RNAseq and WES sequencing reads to human and mouse genomes. [file crc-21-0126-s02.pdf]

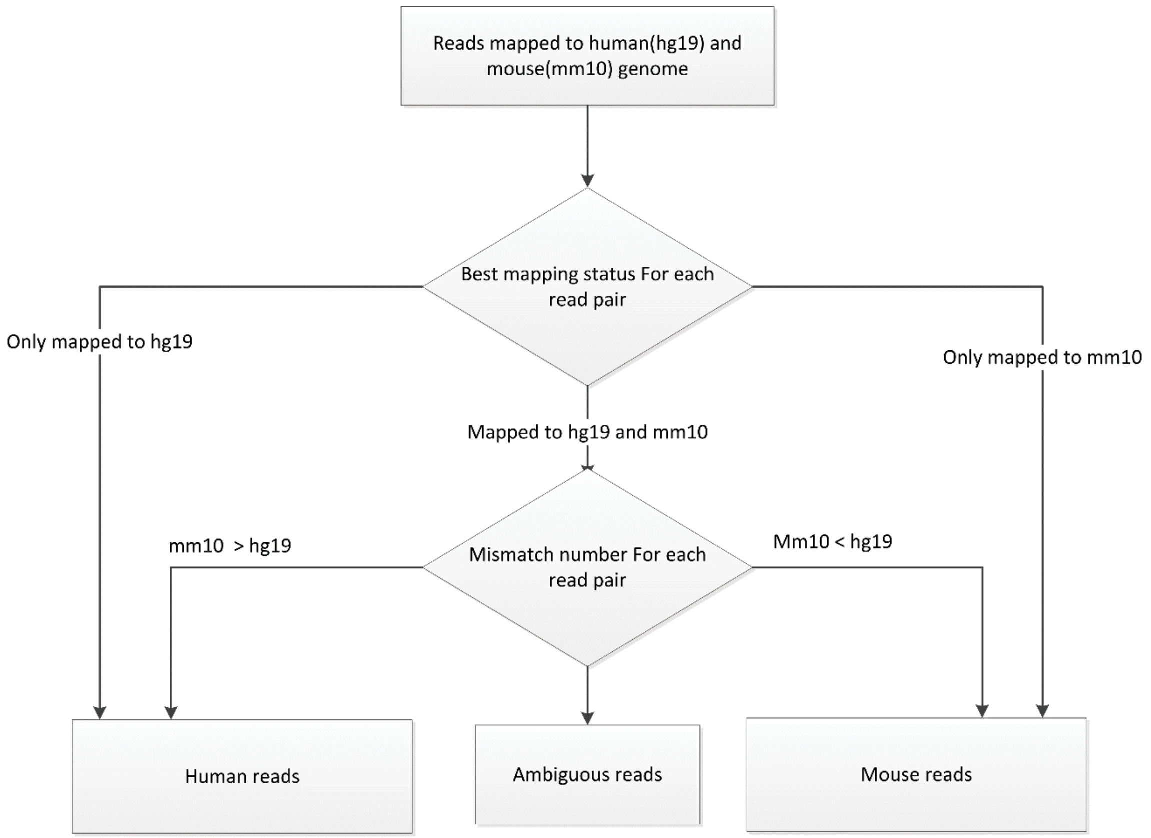

Supplementary Figure 1. Computational workflow for mapping RNAseq and WES sequencing reads to human and mouse genomes. For PDX tumors, the sequencing reads were mapped to human (hg19) and mouse (mm10) reference genomes using STAR (version 2.7.10a) for RNAseq data and BWA (version 0.7.17) for WES data. Reads preferentially mapped to the mouse or human genomes were taken as mouse or human reads, respectively, ambiguous reads were discarded.
