# Supplementary material for: Tumor Purity in Preclinical Mouse Tumor Models
Source: Cancer Res Commun. 2022 May 10;2(5):353–65. doi: 10.1158/2767-9764.CRC-21-0126 (PMC9981214; doi:10.1158/2767-9764.CRC-21-0126)
Supplement: Supplementary Figure 2 — Tumor purity inferred from ESTIMATE score from RNAseq data for human samples. [file crc-21-0126-s03.pdf]

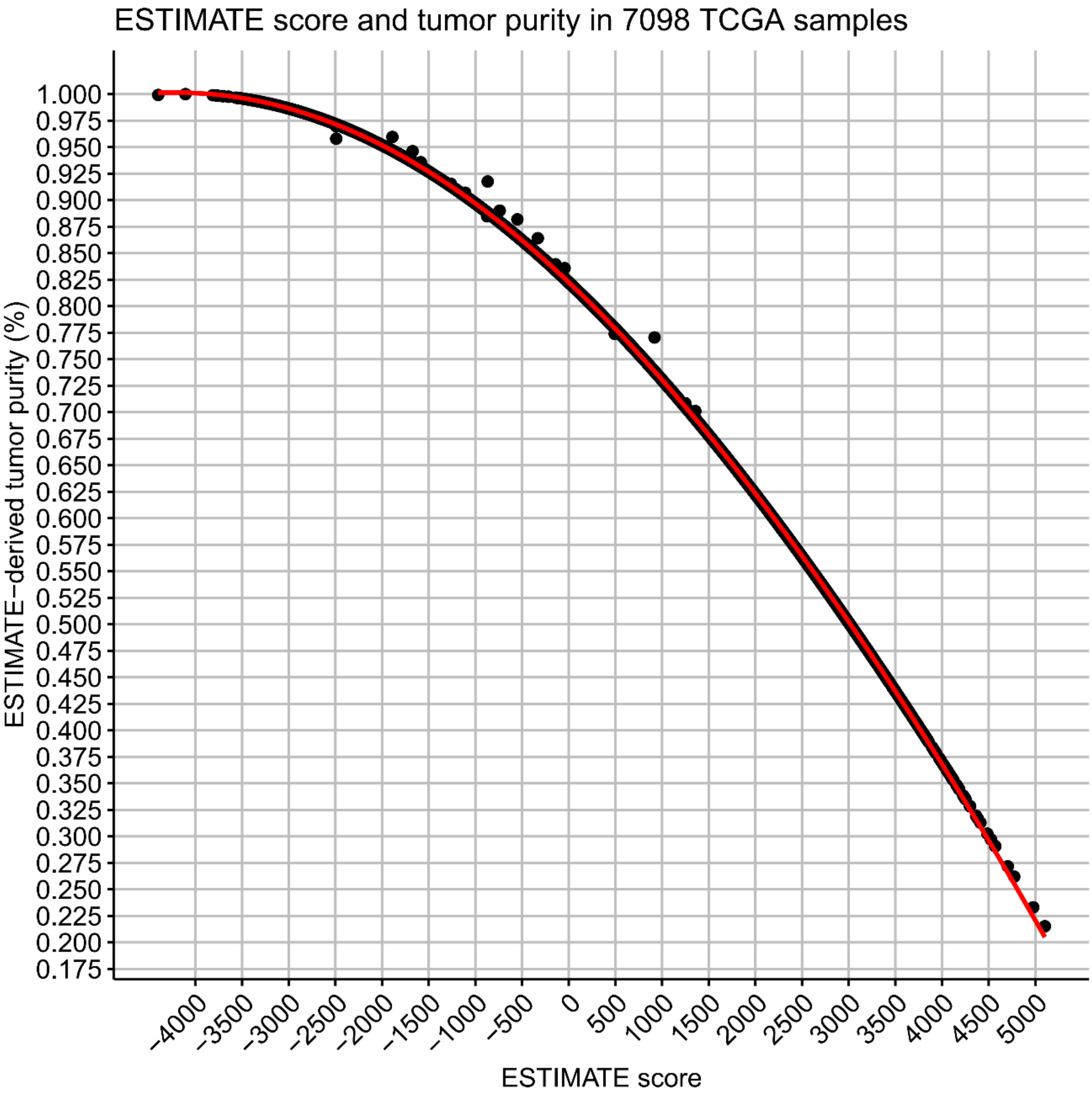

Supplementary Figure 2. Tumor purity inferred from ESTIMATE score from RNAseq data for human samples. A near-perfect non-linear relationship between ESTIMATE score and tumor purity for 7098 TCGA patient tumor samples was established by the `loess` function with default setting in the R software package (version 4.1.1).
