# Supplementary material for: Tumor Purity in Preclinical Mouse Tumor Models
Source: Cancer Res Commun. 2022 May 10;2(5):353–65. doi: 10.1158/2767-9764.CRC-21-0126 (PMC9981214; doi:10.1158/2767-9764.CRC-21-0126)
Supplement: Supplementary Figure 3 — Median variant allelic fraction (VAF) of missense mutations in cancer cell lines and patient tumors. [file crc-21-0126-s04.pdf]

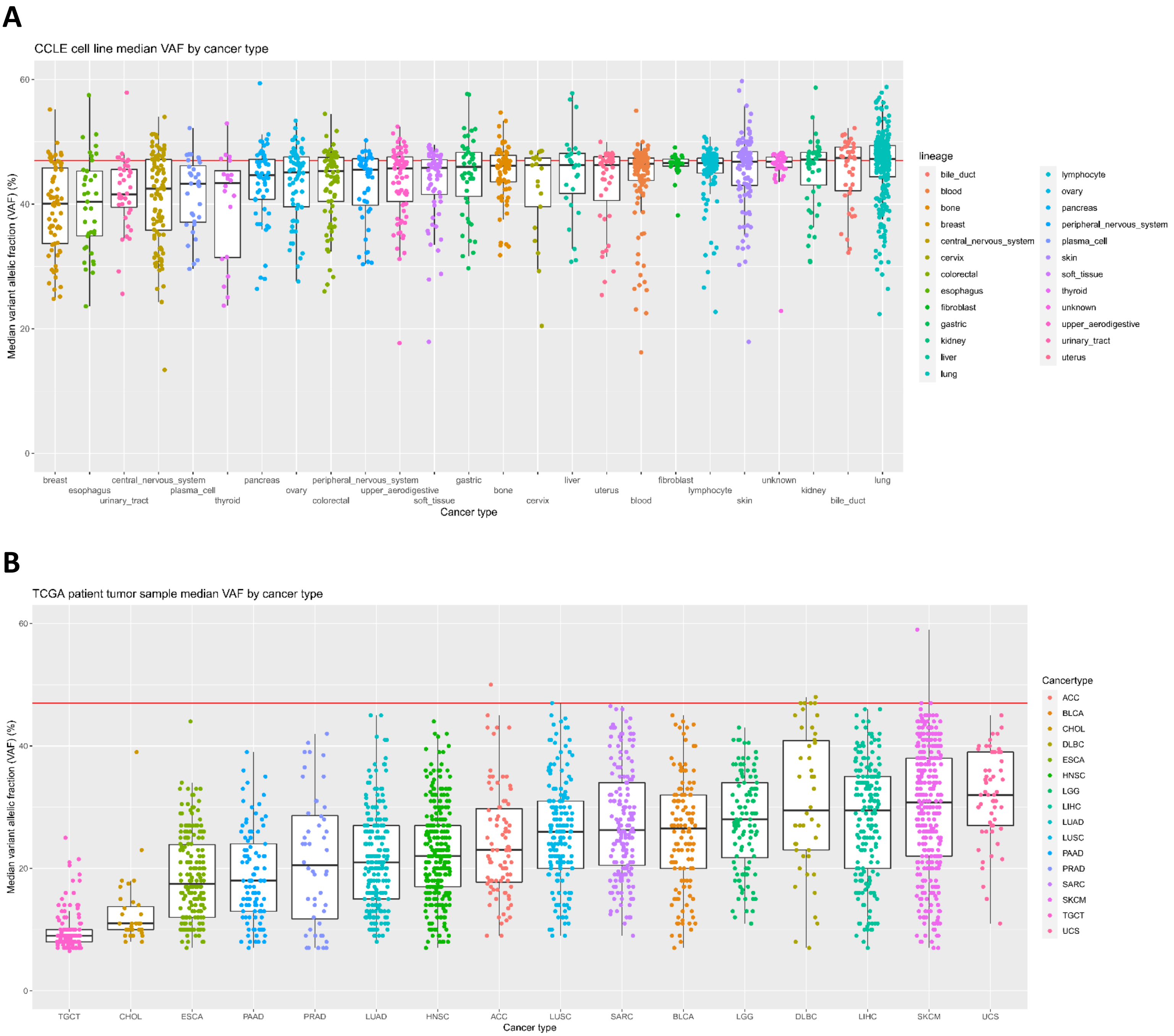

Supplementary Figure 3. Median variant allelic fraction (VAF) of missense mutations in cancer cell lines and patient tumors. (**A**) Median VAF distribution in 1649 cancer cell lines, each with at least 30 missense somatic mutations that have >5% mutation frequency. (**B**) Median VAF distribution in 7098 TCGA patient tumor samples, each with at least 30 missense somatic mutations that have >5% mutation frequency. *Abbreviations*: TGCT, Testicular Germ Cell Tumors; CHOL, Cholangiocarcinoma; ESCA, Esophageal carcinoma; PAAD, Pancreatic adenocarcinoma; PRAD, Prostate adenocarcinoma; LUAD, Lung adenocarcinoma; HNSC, Head and Neck squamous cell carcinoma; ACC, Adrenocortical carcinoma; LUSC, Lung squamous cell carcinoma; SARC, Sarcoma; BLCA, Bladder Urothelial Carcinoma; LGG, Brain Lower Grade Glioma; DLBC, Lymphoid Neoplasm Diffuse Large B-cell Lymphoma; LIHC, Liver hepatocellular carcinoma; SKCM, Skin Cutaneous Melanoma; UCS, Uterine Carcinosarcoma. Cell line mutation data were downloaded from the DepMap project website (version 21Q3), TCGA data were retrieved from cBioportal on Jan 18, 2022.
