# Supplementary material for: Tumor Purity in Preclinical Mouse Tumor Models
Source: Cancer Res Commun. 2022 May 10;2(5):353–65. doi: 10.1158/2767-9764.CRC-21-0126 (PMC9981214; doi:10.1158/2767-9764.CRC-21-0126)
Supplement: Supplementary Figure 4 — The distribution of tumor purity in human cells of 2115 PDX models within passage 10. [file crc-21-0126-s05.pdf]

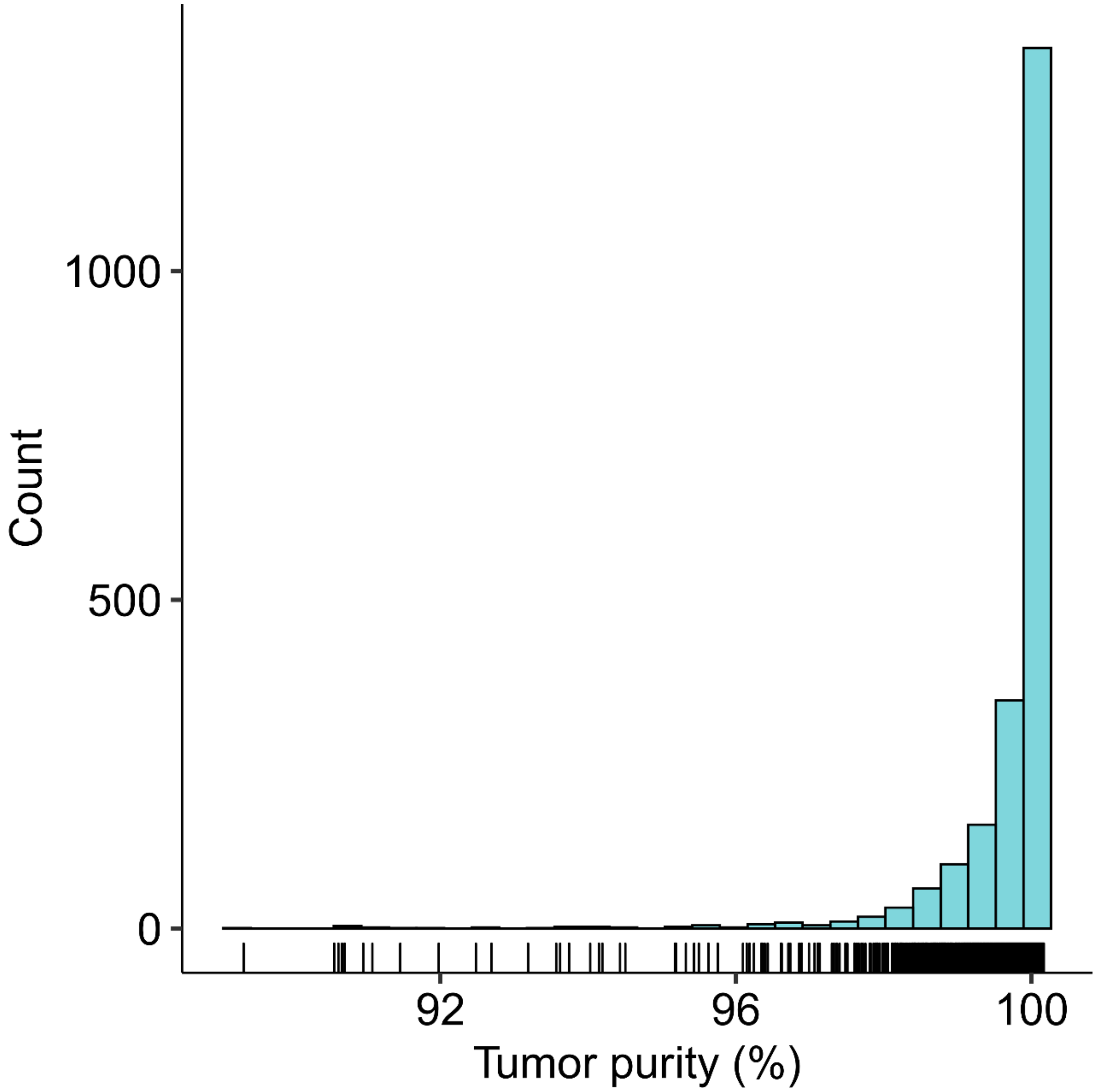

Supplementary Figure 4. The distribution of tumor purity in human cells of 2115 PDX models within passage 10. For each PDX tumor, the ESTIMATE algorithm was applied to human gene expression computed from RNAseq data to obtain an ESTIMATE score that was used by the non-linear function in Fig. S2 to obtain tumor purity.
