# Supplementary material for: Tumor Purity in Preclinical Mouse Tumor Models
Source: Cancer Res Commun. 2022 May 10;2(5):353–65. doi: 10.1158/2767-9764.CRC-21-0126 (PMC9981214; doi:10.1158/2767-9764.CRC-21-0126)
Supplement: Supplementary Figure 5 — Within- and between-PDX tumor purity difference for 18 cancers. [file crc-21-0126-s06.pdf]

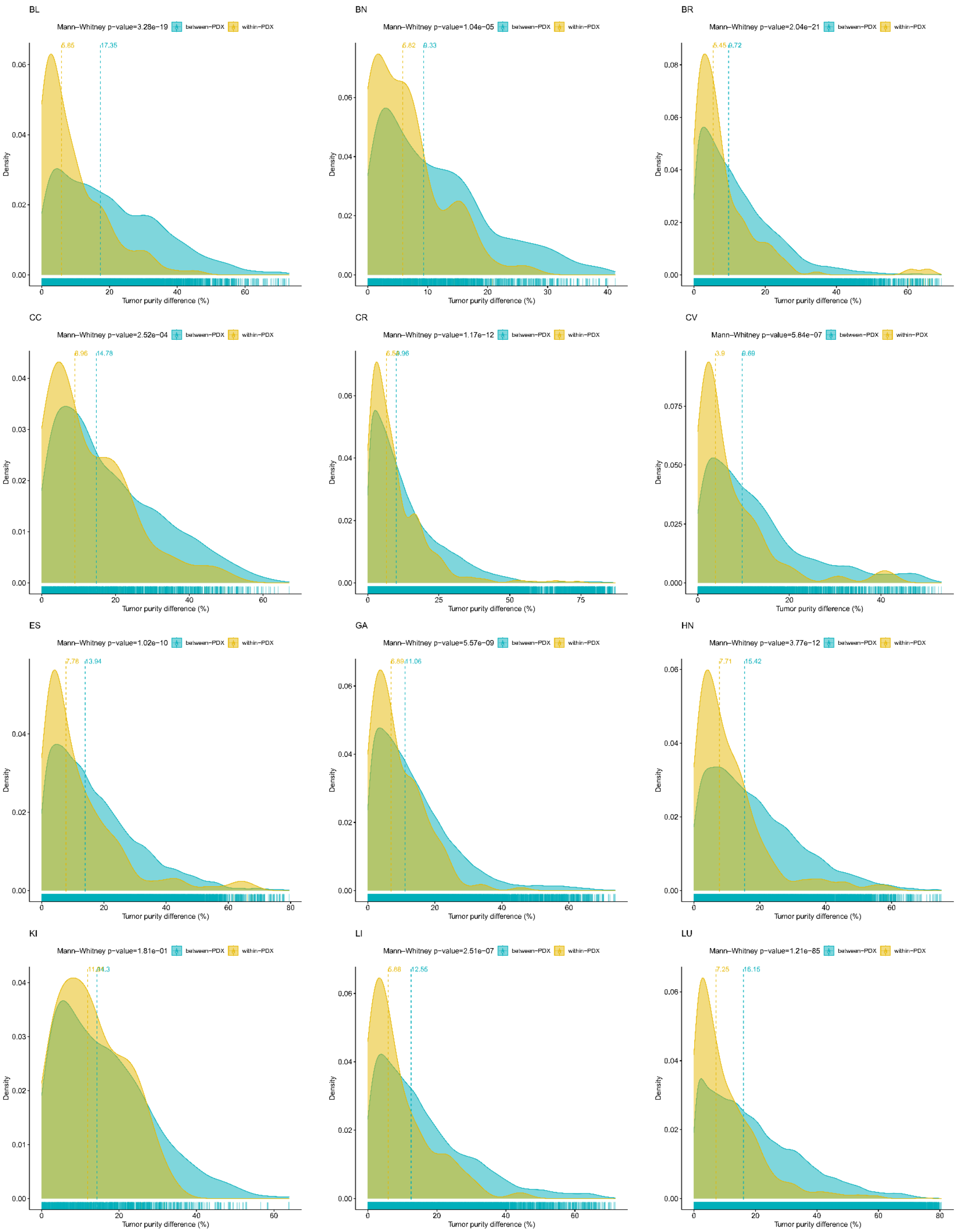

BL
Mann–Whitney p-value=3.28e−19
between−PDX
within−PDX
5.85
17.35
Density
Tumor purity difference (%)
BN
Mann–Whitney p-value=1.04e−05
9.33
BR
Mann–Whitney p-value=2.04e−21
CC
Mann–Whitney p-value=2.52e−04
8.96
14.78
CR
Mann–Whitney p-value=1.17e−12
CV
Mann–Whitney p-value=5.84e−07
3.9
9.69
ES
Mann–Whitney p-value=1.02e−10
7.76
13.94
GA
Mann–Whitney p-value=5.57e−09
11.06
HN
Mann–Whitney p-value=3.77e−12
7.71
15.42
KI
Mann–Whitney p-value=1.81e−01
LI
Mann–Whitney p-value=2.51e−07
5.88
12.55
LU
Mann–Whitney p-value=1.21e−85
7.25
16.15

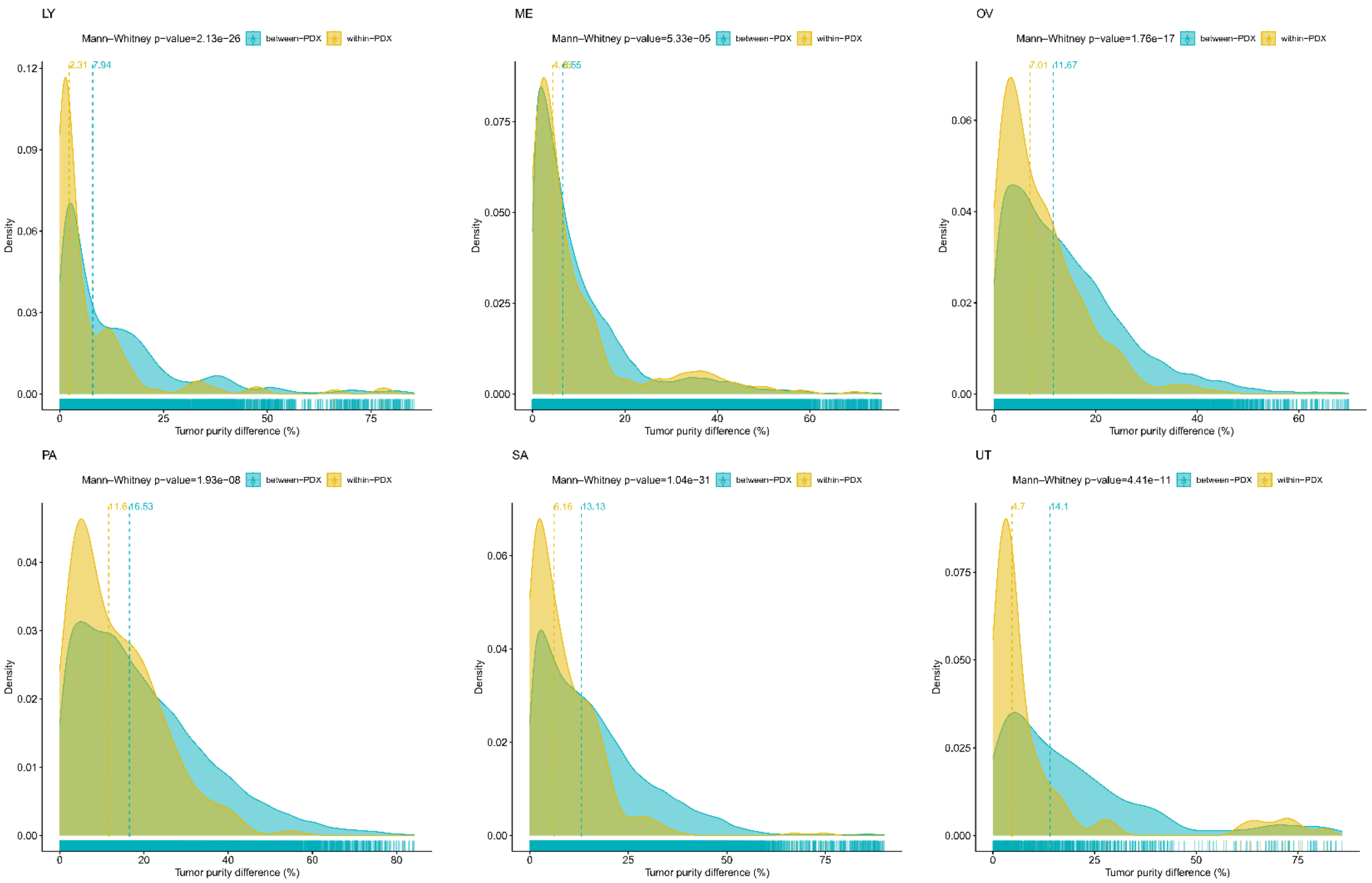

Supplementary Figure 5. Within- and between-PDX tumor purity difference for 18 cancers. *Cancer abbreviations*: BL, bladder; BN, brain; BR, breast; CC, cholangiocarcinoma; CR, colorectal; CV, cervical; ES, esophageal; GA, gastric; HN, head and neck; KI, kidney; LI, liver; LU, lung; LY, lymphoma; ME, melanoma; OV, ovarian; PA, pancreatic; SA, sarcoma; UT, uterine.
