# Supplementary material for: Tumor Purity in Preclinical Mouse Tumor Models
Source: Cancer Res Commun. 2022 May 10;2(5):353–65. doi: 10.1158/2767-9764.CRC-21-0126 (PMC9981214; doi:10.1158/2767-9764.CRC-21-0126)
Supplement: Supplementary Figure 6 — PDX tumor purity change by passage for 8 cancers. [file crc-21-0126-s07.pdf]

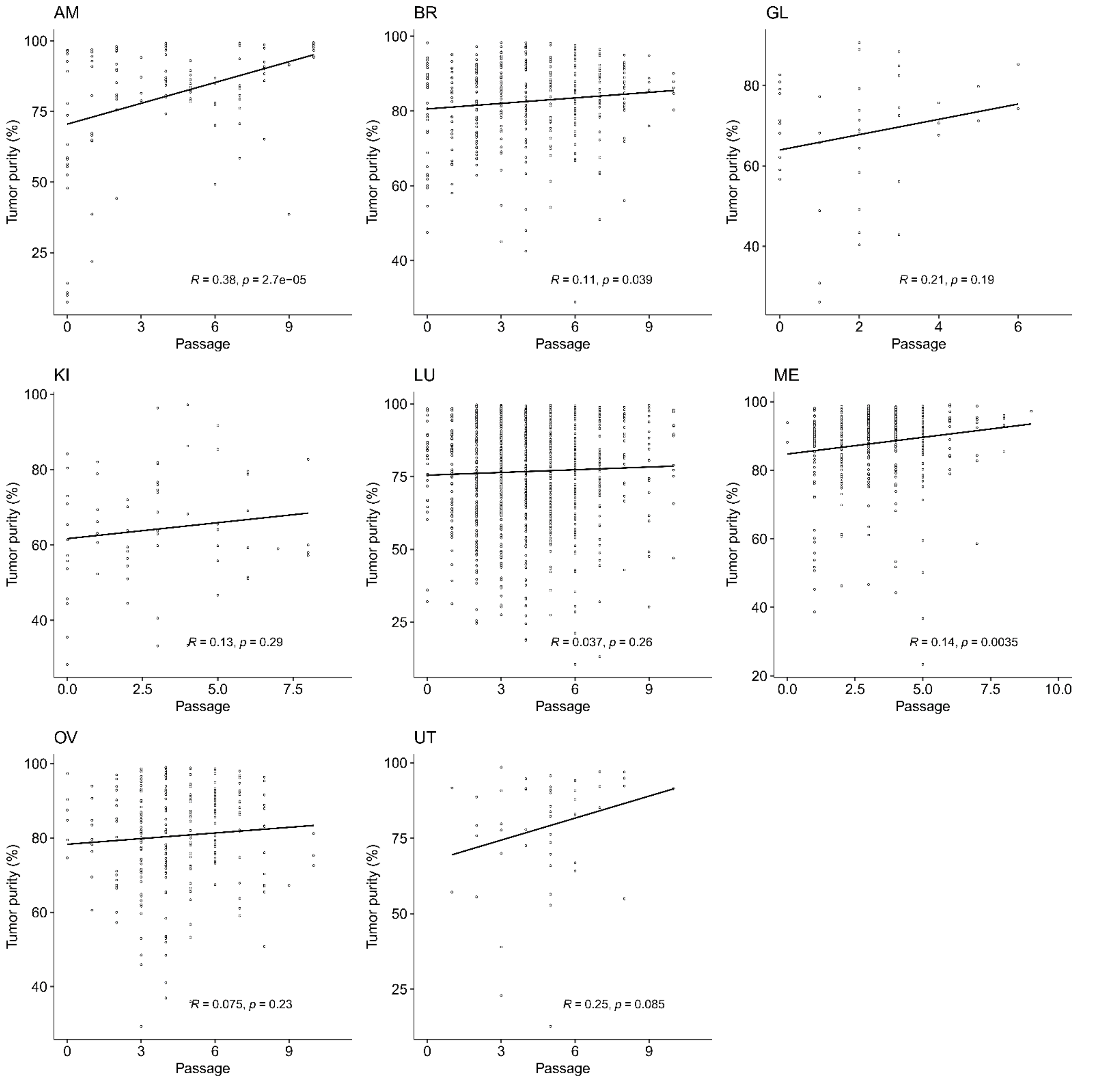

Supplementary Figure 6. PDX tumor purity change by passage for 8 cancers. *Cancer abbreviations*: AM, acute myeloid leukemia; BR, breast; GL, gallbladder; KI, kidney; LU, lung; ME, melanoma; OV, ovarian; UT, uterine.
