# Supplementary material for: Tumor Purity in Preclinical Mouse Tumor Models
Source: Cancer Res Commun. 2022 May 10;2(5):353–65. doi: 10.1158/2767-9764.CRC-21-0126 (PMC9981214; doi:10.1158/2767-9764.CRC-21-0126)
Supplement: Supplementary Figure 7 — The distribution of tumor purity in mouse cells for 2115 PDX models within passage 10. [file crc-21-0126-s08.pdf]

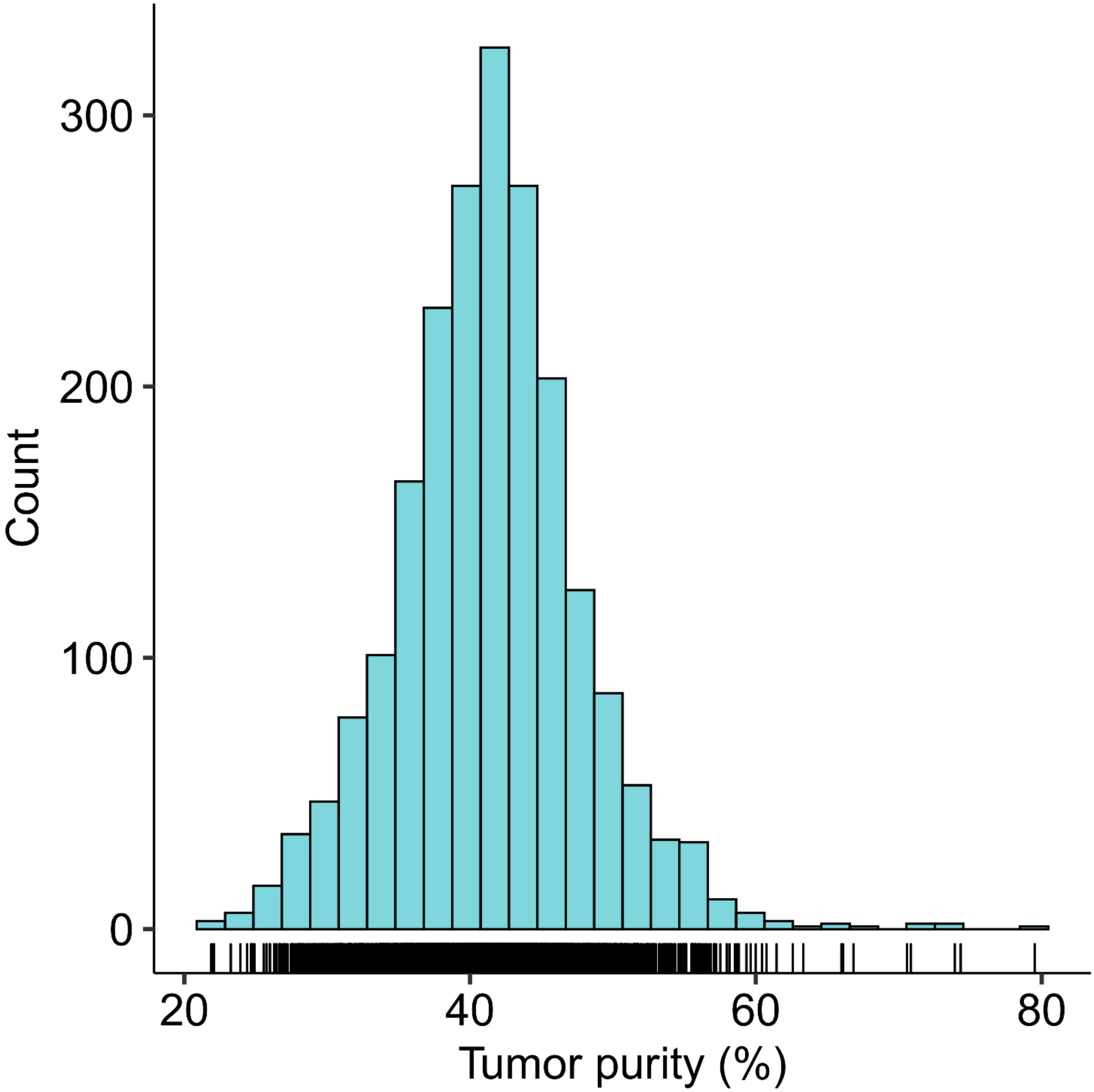

Supplementary Figure 7. The distribution of tumor purity in mouse cells for 2115 PDX models within passage 10. For each PDX tumor, the mouse version of ESTIMATE algorithm was applied to the mouse gene expression, computed from RNAseq data, to obtain an ESTIMATE score that was used by the non-linear function in Fig. S2 to obtain tumor purity.
